# Cornelian Cherry Pulp Has Beneficial Impact on Dyslipidemia and Reduced Bone Quality in Zucker Diabetic Fatty Rats

**DOI:** 10.3390/ani10122435

**Published:** 2020-12-19

**Authors:** Radoslav Omelka, Jana Blahova, Veronika Kovacova, Martina Babikova, Vladimira Mondockova, Anna Kalafova, Marcela Capcarova, Monika Martiniakova

**Affiliations:** 1 Department of Botany and Genetics, Faculty of Natural Sciences, Constantine the Philosopher University in Nitra, 949 74 Nitra, Slovakia; jana.blahova@ukf.sk (J.B.); martina.babikova@ukf.sk (M.B.); vmondockova@ukf.sk (V.M.); 2 Department of Zoology and Anthropology, Faculty of Natural Sciences, Constantine the Philosopher University in Nitra, 949 74 Nitra, Slovakia; vkovacova@ukf.sk; 3 Department of Animal Physiology, Faculty of Biotechnology and Food Sciences, Slovak University of Agriculture in Nitra, 949 76 Nitra, Slovakia; anna.kalafova@uniag.sk (A.K.); marcela.capcarova@uniag.sk (M.C.)

**Keywords:** Cornelian cherry, diet, diabetes mellitus, bone quality, biochemistry, microcomputed tomography, histomorphometry, Zucker diabetic fatty (ZDF) rats

## Abstract

**Simple Summary:**

Fruits of Cornelian cherry (*Cornus mas* L.) are often used as an antidiabetic supplement mainly due to their hypoglycemic properties. A very important aspect and secondary complication of type 2 diabetes mellitus (T2DM) represents diabetic bone disease. In our study, the impacts of *Cornus mas* pulp on lipid profile and bone quality parameters were evaluated in Zucker diabetic fatty (ZDF) rats as a well-matched T2DM animal model. We demonstrated, for the first time, that Cornelian cherry pulp could be used as a potential therapeutic agent to alleviate T2DM-reduced bone quality and impaired bone health. Moreover, the hypolipidemic effect of this fruit was also confirmed in our study.

**Abstract:**

Cornelian cherry (*Cornus mas* L.) is a medicinal plant with a range of biological features. It is often used as a nutritional supplement in the treatment of diabetes mellitus. Our study was aimed to first investigate the effects of Cornelian cherry pulp on bone quality parameters in Zucker diabetic fatty (ZDF) rats. Moreover, lipid-lowering properties of this fruit were also evaluated. Adult rats (n = 28) were assigned into four groups of seven individuals each: L group (non-diabetic lean rats), C group (diabetic obese rats), and E1 and E2 groups (diabetic obese rats receiving 500 and 1000 mg/kg body weight of Cornelian cherry pulp, respectively, for 10 weeks). Significantly lower levels of triglyceride, total cholesterol and alkaline phosphatase activity were determined in the E2 group versus the C group. A higher dose of *Cornus mas* also had a beneficial impact on femoral weight, cortical bone thickness, relative volume of trabecular bone and trabecular thickness. We observed elevated density of Haversian systems and accelerated periosteal bone apposition in both treated groups (E1 and E2). Our results clearly demonstrate that Cornelian cherry pulp has a favorable effect on lipid disorder and impaired bone quality consistent with type 2 diabetes mellitus in a suitable animal model.

## 1. Introduction

Type 2 diabetes mellitus (T2DM) is a chronic endocrine disorder with increasing prevalence worldwide. It is characterized by insulin deficiency or ineffective insulin production by the pancreas, resulting in persistent hyperglycemia [1,2]. T2DM negatively affects glucose transport into the liver, muscle cells and fat cells [3]. These conditions also adversely influence the skeletal system via diabetic bone disease. Harmful impacts are associated with bone strength, bone remodeling and stem cell differentiation and lead to varied bone mineral density (BMD) and changed bone structure [4]. Hyperglycemia can suppress BMD through altered osteoblast gene expression, reduced osteoblast function and number, higher oxidative stress and adipogenesis, downregulation of vitamin D receptors and increased production of glycation end-products (AGEs) [5]. AGEs inhibit bone remodeling and indirectly attenuate osteoblast activity and apoptosis of osteocytes.

In general, bone remodeling is a major mechanism for maintaining a healthy skeleton in adults, and bone remodeling dysfunction may contribute to bone loss and/or impaired bone quality [6,7]. Bone turnover markers, which reflect the bone resorption and formation processes, are usually reduced in individuals with T2DM (mainly osteocalcin as bone formation marker and C-terminal telopeptide of type I collagen as bone resorption marker) [8]; however, not all studies yielded consistent findings. Some reports found no differences in the bone turnover markers or even their increased levels in T2DM [9]. The differences between various studies may be explained by changes in metabolic status, diabetes duration and treatment at the time of the measurement [10]. Despite conflicting findings, the evidence seems to point towards a suppression of bone remodeling in diabetes and also in the pre-diabetic state [8].

The association between T2DM and BMD is also controversial. Most studies showed decreased or unchanged BMD; however, some others revealed increased BMD [11,12,13]. Another diabetic complication contributing to detrimental bone effects is microangiopathy. Impaired vascularity and T2DM-enhanced inflammation can cause improper distribution of nutrients, oxygen, hormones or growth factors to the bone cells. It can lead to impaired bone healing [14,15], loss of bone strength and increased bone fragility [16].

Zucker diabetic fatty (ZDF) rats are considered to be an appropriate animal model of type 2 diabetes mellitus, with the spontaneous mutation on chromosome 5, which encodes leptin receptors. This mutation causes obesity, glucose intolerance, hyperglycemia, hyperlipidemia and hyperinsulinemia [17,18]. ZDF rats show lower trabecular bone volume and trabecular number, higher trabecular separation and cortical porosity, improper vascularization [19], and decreased osteoblast differentiation and mineral capacity [15].

Active substances extracted from plants can be useful to prevent or support the therapy of various pathological conditions, including diabetes mellitus [20]. Generally, plant products have high nutritional value and possess beneficial impacts for health. Cornelian cherry (*Cornus mas* L.) is a flowering plant that belongs to Cornaceae family, with occurrence in Southern and Central Europe, as well as Western Asia. Cornelian cherry fruit is used for food, syrup and jam production, and in the fermented form, it is used to make alcoholic beverage [21,22]. It is rich in proteins, vitamins, antioxidants, mineral matters [23,24,25], polyphenols (e.g., anthocyanins, flavonoids, catechins and tannins) and other essential supplements [22]. All of these compounds have antioxidant, antimicrobial, hypolipidemic, hyperinsulinemic [26], anti-inflammatory and anticancer properties and also offer positive support to the nervous and cardiovascular systems [25].

The main aim of this in vivo study was to first analyze the effect of Cornelian cherry pulp on bone quality parameters in ZDF rats, since the impact of this fruit as a potential therapeutic agent to alleviate T2DM-reduced bone quality has not been investigated yet. Moreover, lipid-lowering properties of *Cornus mas* were also evaluated.

## 2. Materials and Methods

### 2.1. Sample Preparation

The experiment was authorized under the number 2288/16-221 by the Ethical Committee and the State Veterinary and Food Administration of the Slovak Republic. Cornelian cherries were supplied by the Institute of Biodiversity Conservation and Biosafety of the Slovak University of Agriculture in Nitra (Slovakia). The fresh ripe fruits were washed, isolated from the stones, crushed and stored at −20 °C. Aliquots of fruits, homogenized in distilled water, were used in the experiment.

### 2.2. Animals

Adult ZDF rats (n = 28) were obtained from the Institute of Experimental Pharmacology and Toxicology, Slovak Academy of Sciences. The experiment was performed at the Slovak University of Agriculture in Nitra. The rats were housed in pairs, in cages of 1500 U Eurostandard Type IV S (Tecniplast, Buguggiate, Italy), with 12 h light/12 h dark cycle, at a room temperature of 22 ± 2 °C, with free access to food and drinking water. The complete rat feed mixture KKZ-P/M (register number 6147, Dobra Voda, Slovakia; composition of the mixture is given in the study by Capcarova et al. [27]) was used for feeding. The animals were divided into 4 groups: L group (n = 7) consisted of non-diabetic lean rats and served as a negative control; C group (n = 7) included diabetic obese rats which were considered as a positive control; and E1 (n = 7) and E2 (n = 7) groups consisted of diabetic obese rats receiving 500 and 1000 mg/kg body weight (bw) of Cornelian cherry pulp, respectively, for 10 weeks. Groups E1 and E2 received the exact dose of *Cornus mas* directly into the stomach, every day, using sterile oral rodent gavage (Instech, Plymouth, MA, USA), whereas groups L and C received distilled water by the same way. The doses of *Cornus mas* were selected based on the study by Capcarova et al. [27]. The animals were sacrificed by intraperitoneal injection of xylazine/zoletil cocktail and all samples were collected after deep anesthesia.

### 2.3. Biochemistry

Blood glucose levels were determined with a FreeStyle Optium Neo Glucose and Ketone monitoring system (Abbott Diabetes Care Ltd., Maidenhead, UK), using test strips (FreeStyle, Abbott Diabetes Care Ltd., Maidenhead, UK). The levels of total cholesterol, LDL cholesterol, HDL cholesterol, triglyceride and alkaline phosphatase (ALP) were measured by a Biolis 24i Premium analyzer (Tokyo Boeki MediSys Inc., Tokyo, Japan), with commercially available kits (Randox Laboratories Ltd., Crumlin, UK).

### 2.4. Macroscopic Measurements

Prior to 3D (microcomputed tomography) and 2D (histomorphometry) imaging, the femoral bones (n = 56) were weighed, and their lengths were measured. Furthermore, the total body weight of all animals was also determined.

### 2.5. Microcomputed Tomography

Microcomputed tomography (microCT, μCT 50, Scanco Medical, Brüttisellen, Switzerland) was used to designate selected quantitative 3D parameters of cortical bone (relative bone volume, relative bone volume with marrow cavity, BMD, cortical bone thickness and bone surface) and trabecular bone sections (relative bone volume, trabecular number, trabecular thickness, BMD and bone surface). The measurement parameters and scanning regions of interest were the same as those in the study by Omelka et al. [28].

### 2.6. Histomorphometry

The femurs were cut at the diaphysis, and the sections were fixed, dehydrated, degreased and embedded according to the method defined by Martiniaková et al. [29]. A sawing microtome (Leitz 1600, Leica, Wetzlar, Germany) was used to prepare thin sections, as previously described [30]. The qualitative 2D characteristics were recorded according to Enlow and Brown [31] and de Ricqlés et al. [32]. Measured parameters included area of primary osteons’ vascular canals, area of Haversian canals and area of Haversian systems. They were determined by Motic Images Plus 2.0 ML software (Motic China Group Co., Ltd., Nanjing, China).

### 2.7. Data Analysis

Statistical analysis was conducted by using SPSS Statistics 26.0 software (IBM, New York, NY, USA). The data were expressed as mean ± standard deviation. Significant differences in parameters investigated were detected by ANOVA, with post hoc (Games-Howell and/or Tukey’s) tests. A *p* value less than 0.05 was considered to be statistically significant.

## 3. Results

### 3.1. Biochemistry

**Figure 1:**
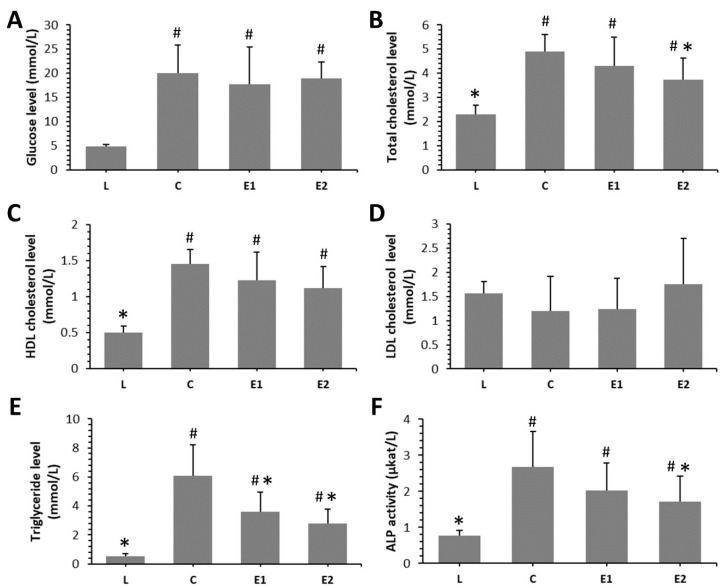
Biochemical parameters examined (**A**–**F**) in Zucker diabetic fatty (ZDF) rats from the L group (non-diabetic lean rats), C group (diabetic obese rats), and E1 and E2 groups (diabetic obese rats receiving 500 and 1000 mg/kg body weight of Cornelian cherry pulp, respectively, for 10 weeks). HDL cholesterol—high-density lipoprotein cholesterol; LDL cholesterol—low-density lipoprotein cholesterol; ALP—alkaline phosphatase. * Significant differences versus C group (*p* < 0.05). # Significant differences versus L group (*p* < 0.05).

Significantly lower levels of triglyceride were determined in both the E1 and E2 groups versus the diabetic control one (C group). In addition, decreased total cholesterol level and ALP activity were recorded in the E2 group, in comparison with the C group. Nevertheless, the blood glucose level did not change significantly in the treated groups against the C group. Significant changes in all parameters investigated were observed between lean rats (L group) and those from the C group, as well as between treated groups (E1 and E2) versus L group. The results are summarized in Figure 1A–F.

### 3.2. Macroscopic Measurements

**Figure 2:**
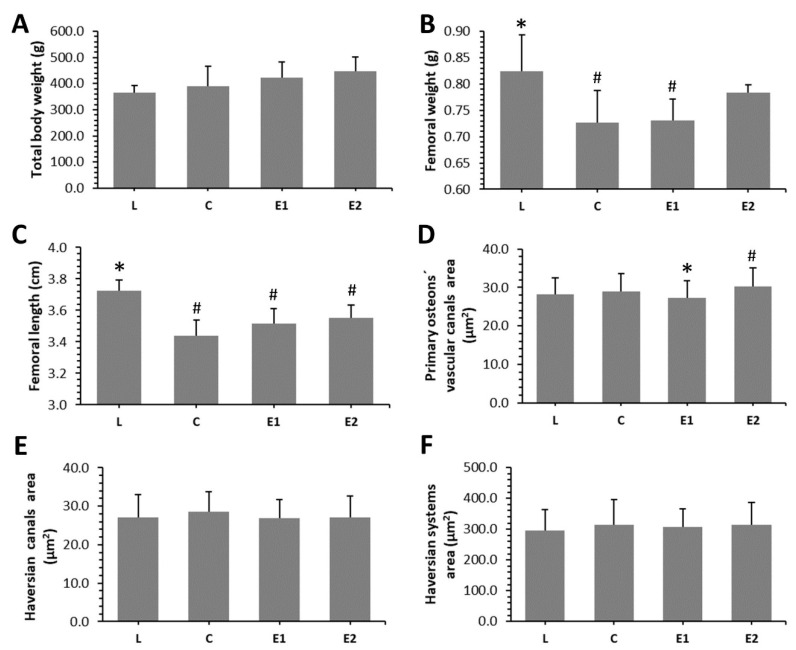
Macroscopical (**A**–**C**) and histomorphometrical (**D**–**F**) parameters examined in ZDF rats from L, C, E1 and E2 groups * Significant differences versus C group (*p* < 0.05). # Significant differences versus L group (*p* < 0.05).

Our results showed non-significant impact of *Cornus mas* pulp on the total body weight of ZDF rats. On the contrary, significantly decreased values for femoral weight and length were observed in C and E1 groups, when compared to the L group. No significant changes in femoral weight were determined between the L and E2 groups, suggesting a positive impact of a higher dose of Cornelian cherry on this parameter. Significantly lower values for femoral length were recorded in the treated groups versus the lean control one. The results are shown in Figure 2A–C.

### 3.3. Microcomputed Tomography

**Figure 3:**
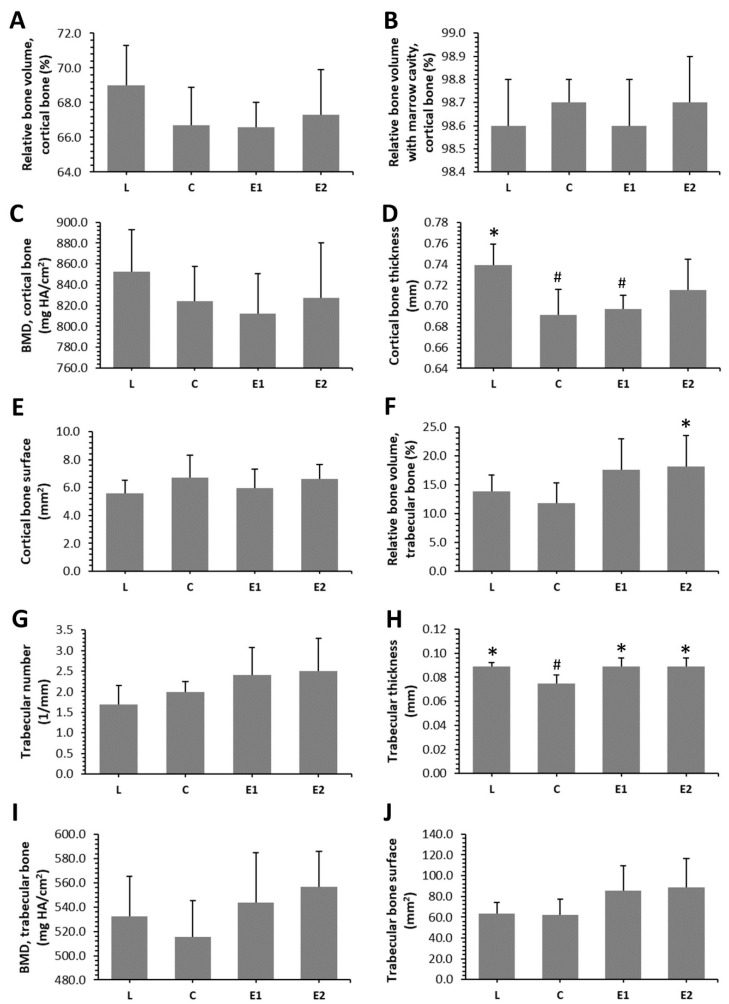
Parameters examined by microcomputed tomography in cortical (**A**–**E**) and trabecular (**F**–**J**) bone regions in ZDF rats from L, C, E1 and E2 groups. BMD—bone mineral density; HA—hydroxyapatite. * Significant differences versus C group (*p* < 0.05). # Significant differences versus L group (*p* < 0.05).

Microcomputed tomography revealed that treatment with Cornelian cherry had an insignificant effect on the relative volume of cortical bone, BMD and cortical bone surface. On the other hand, significantly decreased cortical bone thickness was determined in the C and E1 groups, as compared to the L group. No significant changes between the L and E2 groups point to a beneficial impact of a higher dose of *Cornus mas* on cortical bone thickness. The results are summarized in Figure 3A–E.

**Figure 4:**
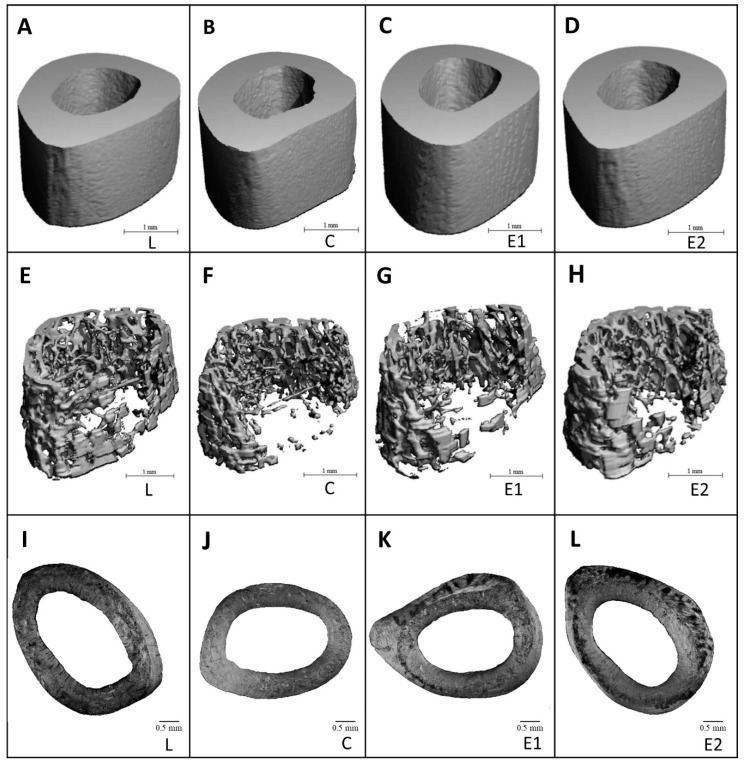
Representative 3D images of cortical (**A**–**D**) and trabecular bone regions (**E**–**H**), and representative 2D images of cortical bone (**I**–**L**) in ZDF rats from L, C, E1 and E2 groups.

Representative reconstructed 3D images of cortical bone are illustrated in Figure 4A–D. A significantly increased relative volume of trabecular bone was observed in the E2 group versus the C group. Significantly higher trabecular thickness was determined in both treated groups, in comparison with the C group. No significant impact of Cornelian cherry administration on trabecular number, trabecular bone surface and BMD was recorded. The data are summarized in Figure 3F–J. Representative reconstructed 3D images of trabecular bone are illustrated in Figure 4E–H.

### 3.4. Histomorphometry

Endosteal and periosteal surfaces of the compact bone were composed of non-vascular bone tissue in all groups. Primary vascular radial bone tissue was observed near the endosteum and in the middle of substantia compacta. However, a higher density of Haversian systems was recorded in the E1 and E2 groups versus the C group. In addition, intensive periosteal bone apposition was determined in both treated groups (Figure 4I–L). In total, 796 primary osteons’ vascular canals, 337 Haversian canals and 337 Haversian systems were measured. We determined a significantly decreased area of primary osteons’ vascular canals in the E1 versus the C and E2 groups. Interestingly, the area of these canals was higher in the E2 group when compared to the L group. On the contrary, the area of Haversian canals and Haversian systems was not affected by Cornelian cherry treatment. The data are summarized in Figure 2D–F.

## 4. Discussion

A previous study [27] demonstrated that administration of *Cornus mas* at the same doses which were used in our research caused significant reduction of water intake in ZDF rats versus the diabetic control ones. However, feed intake in diabetic control group did not differ significantly when compared to both treated groups. Moreover, ZDF rats from the E1 and E2 groups had significantly decreased blood glucose level versus the C group only in the pre-diabetic state (fifth to seventh week). At the end of the experiment (after 10 weeks), blood glucose did not reach significant values among the C, E1 and E2 groups, thus corresponding to our findings. We determined insignificantly lower blood glucose level in both treated groups versus the C group. Capcarova et al. [27] also observed that the rats from the E1 and E2 groups exhibit similar values of insulin than those from the diabetic control group. Therefore, other biochemical parameters that would reflect lipid-lowering properties of Cornelian cherry in diabetes were evaluated in our study. Enhanced levels of triglyceride, total cholesterol, HDL cholesterol, LDL cholesterol and ALP activity were recorded in the diabetic control versus the lean control group, which is consistent with the findings of Pang et al. [33]. Our results revealed that a higher dose of *Cornus mas* reduces triglyceride, total cholesterol levels and ALP activity. It can be related to its hypotriglyceridemic and hypocholesterolemic properties, as well as its protective effect against liver and bone disease. Soltani et al. [24] also identified a decreased value of triglyceride in T2DM patients receiving the fruit extract of *Cornus mas*. In alloxan-induced diabetic rats, hydroalcoholic fruits of Cornelian cherry also lowered triglyceride level [34]. In the study by Asgary et al. [21], reduced values of triglyceride and ALP activity were observed in diabetic rats after *Cornus mas* administration, as well. In general, ALP is considered as a good sensitive marker to access bone formation. However, elevated ALP in T2DM can indicate not only bone failure but also liver disease, or both. For that reason, it would be necessary to investigate the levels of other bone formation and bone resorption markers in the blood of ZDF rats to obtain more accurate results.

Cornelian cherry treatment insignificantly increased the total body weight of ZDF rats in our study. One of diabetes symptoms is a decrease in body weight caused by insulin resistance and impairment in whole-body glucose uptake. Dayar et al. [35] also observed non-significant impact of wild type Cornelian cherries administration (5 g/kg/day for six weeks) on the body weight in obese Zucker rats. Our study seems to be the first evaluating the effect of Cornelian cherry pulp on femoral weight and length. No significant changes in femoral weight were determined between L and E2 groups which could be consistent with a positive impact of higher dose of this fruit on bone weight. Generally, one of the most important components of Cornelian cherry fruit are anthocyanins. Van der Heijden et al. [36] did not find significant changes in the total body weight, weight of liver and adipose tissue in obese mice receiving high-energy diet with 36% extract of anthocyanins. Neither Shimizu et al. [37] revealed a significant impact of anthocyanins-rich bilberry extract (500 mg/kg bw/day for eight weeks) on the total body weight and weight of uterus in ovariectomized (OVX) rats. According to Damiano et al. [38], the body weight was significantly increased in ZDF rats compared with controls, whereas red orange and lemon extract rich in anthocyanins in ZDF rats significantly restored these values.

We demonstrated, for the first time, that higher dose of *Cornus mas* has also favorable effect on femoral weight, cortical bone thickness, relative volume of trabecular bone and trabecular thickness. In addition, elevated trabecular thickness was also observed in E1 versus C group. Thus, the increase in trabecular volume was mostly not due to an increase in their number but to their thickness.

Higher density of Haversian systems and accelerated periosteal bone apposition were determined in our treated groups. Through Haversian intracortical remodeling, fatigue microdamages are repaired (targeted remodeling), but it is also present at locations where microdamage do not occur (non-targeted remodeling) [39]. According to Bell et al. [40], enhanced density of smaller Haversian systems decreases microdamage propagation. In general, larger Haversian systems are more vulnerable to microfractures, and therefore undertake targeted remodeling [39,41]. In agreement with our results, Cornelian cherry pulp has strengthened the bone through both mechanisms Haversian remodeling and periosteal bone modeling. However, additional analyses related to mechanical properties of the cortical bone (e.g., maximum displacement, fracture load, stiffness and energy absorption) should be performed in order to confirm these results.

We determined lower area of primary osteons’ vascular canals in E1 versus C, E2 groups. However, the area of these canals was higher in E2 group against lean control one. It is known that polyphenolic compounds in Cornelian cherry support angiogenesis, function of endothelium, and enhance the proliferation and migration of cells in the blood vessel [42,43]. We have found that the effect of *Cornus mas* on blood vessels in vascular canals of primary osteons depends on the dose used. On the contrary, Cornelian cherry treatment did not affect the size of Haversian canals and Haversian systems. The vascular structures in Haversian canals show typical capillary properties and are often paired. In general, they are fenestrated, lined with partial layer of endothelial cells and bounded by a continual thick basal membrane that restricts the transport of ions across the capillary [44]. It is known that Haversian system is the classic result of the process of bone remodeling. Bone remodeling is closely associated with vascular remodeling that means blood vessels in Haversian canals are able to modify its structure unlike those present in primary osteons.

The most important causes of bone damage and reduced bone strength, resulting from T2DM-related chronic hyperglycemia, are oxidative stress, production of reactive oxygen species (ROS) and AGEs, inflammation [45]. According to our results, Cornelian cherry pulp had favorable effect on impaired bone quality consistent with T2DM. Given the documented chemical composition and pharmacological activities of *Cornus mas*, it is likely that the mechanism of its action interferes with all aforementioned processes. The main anthocyanin compounds of *Cornus mas* include cyanidin 3-O-galactoside and pelargonidin 3-O-galactoside. Quercetin 3-O-glucuronide is the major flavonoid constituent, followed by kaempferol 3-O-galactoside. Among flavanols, catechin is predominant in fruits [46]. Generally, anthocyanins decrease the blood glucose level and peripheral insulin resistance in animal models of diabetes [47]. We suppose that improvement of blood glucose levels in both E1, E2 groups only in pre-diabetic state could have beneficial effects on bone deterioration in ZDF rats at the end of the experiment, as it is documented by our results from other biochemical parameters, microcomputed tomography and histomorphometry. The anthocyanins and other antioxidant components also inhibit oxidative stress and stress-sensitive signaling pathways. They have been proposed to attenuate ROS, especially by the ability to act as reducing agents in the electron-transfer reaction pathway [48]. It has been documented that the number of hydroxyl groups present on the glycosylated B-ring structure of anthocyanins is associated with their scavenging ability, and may influence their antioxidant capacity. According to Dzydzan et al. [47], the administration of Cornelian cherry extracts also decreased AGEs level in blood plasma which is consistent with the fact that polyphenols are able to inhibit AGEs formation. Moreover, polyphenols exhibit anti-inflammatory activity through peroxisome proliferator-activated receptor alpha (PPARα) expression in the liver and are associated with a decrease in triglyceride and pro-inflammatory cytokines levels [46]. However, recent data suggest that polyphenols can exert their beneficial effects by a compendium of other mechanisms, such the activation of transcription factors involved in antioxidant responsive capacity, metal chelating, and their capacity to bind to several proteins and thus impacting cellular homeostasis [49]. In addition, polyphenols have been shown to protect bone health through the modulation of osteoblastogenesis, osteoclastogenesis and osteoimmunological action [50].

## 5. Conclusions

We demonstrated, for the first time, that Cornelian cherry pulp could be used as a food supplement to alleviate T2DM-reduced bone quality and impaired bone health. Administration of this fruit positively influenced femoral weight, cortical bone thickness, relative volume of trabecular bone, trabecular thickness, Haversian remodeling and periosteal bone modeling. Moreover, hypolipidemic effect of *Cornus mas* was also confirmed in our study, using ZDF rats as an appropriate animal model.
